# Structure and Properties of Alloys Obtained by Aluminothermic Reduction of Deep-Sea Nodules

**DOI:** 10.3390/ma14030561

**Published:** 2021-01-25

**Authors:** Pavel Novák, Nguyen Hong Vu, Lucie Šulcová, Jaromír Kopeček, František Laufek, Alisa Tsepeleva, Petr Dvořák, Alena Michalcová

**Affiliations:** 1 Department of Metals and Corrosion Engineering, University of Chemistry and Technology, Prague, Technická 5, 166 28 Prague 6, Czech Republic; vun@vscht.cz (N.H.V.); sulcoval@vscht.cz (L.Š.); tsepelel@vscht.cz (A.T.); dvorakp@vscht.cz (P.D.); michalca@vscht.cz (A.M.); 2 Institute of Physics of the ASCR, v. v. i., Na Slovance 2, 182 21 Prague 8, Czech Republic; kopecek@fzu.cz; 3 Czech Geological Survey, Geologická 6, 152 00 Prague 5, Czech Republic; frantisek.laufek@geology.cz

**Keywords:** manganese alloy, deep-sea nodules, aluminothermy

## Abstract

This paper brings an innovative processing route of manganese deep-sea nodules, which results in completely new grades of alloys. Deep-sea nodules were processed by aluminothermic method without the extraction of individual elements, producing complexly alloyed manganese-based “natural alloys”. Three levels of the amount of aluminum were used for the aluminothermic reduction, and hence the alloys differ strongly in the amount of aluminum, which has a significant effect on their phase composition. The alloys have very high wear resistance, comparable with tool steel. The disadvantage of low-aluminum alloy is the susceptibility to local thermal cracking during friction, which occurs especially in the case of a dry sliding wear against the static partner with low thermal conductivity.

## 1. Introduction

Polymetallic deep-sea nodules represent the untapped sources of valuable base metals such as Mn, Ni, Cu, and Co, as well as trace amounts of Zn, Mo, Ti, V, Zr, Si, and Al, and also rare earth metals (REEs) [1,2,3]. Found predominantly on the seabed of the world oceans, the nodules are rock concretions of different shapes and sizes ranging from several millimeters to about 30 cm at water depths of approximately 3500 to 6500 m [2,4]. The Clarion–Clipperton Fracture Zones (CCZ) in the Pacific Ocean are the area of greatest economic interest with the total amount of manganese, nickel, and cobalt contained in the nodules exceed to that of terrestrial reserves [2,5]. On average, the nodules from CCZ contain 29.1% Mn, 5.4% Fe, 1.29% Ni, 1.19% Cu, and 0.23% Co [6]. The major crystalline phases in the nodules are Todorokite, (mixed oxides of Mn, Mg, Ca, Na, and K), Buserite (hydrated oxides of Mn and Na), Birnessite ((Na_7_Ca_3_)Mn_70_O_140_·28H_2_O) and Vernadite (δ-MnO_2_) or Manganosite (MnO) [1,2,3,7].

Previous research has focused mainly on extraction of Cu, Ni, and Co from the polymetallic nodules; however, Mn extraction has gained more attention recently [6,8,9,10,11].

Since the valuable metals in nodules are present as an integral part of iron and manganese oxides, it is essential to release them by disintegrating the matrix of iron and manganese oxide lattice in order to achieve high recovery of metals. This can be achieved by subjecting the nodules to a reducing condition. In principle, both pyrometallurgical and hydrometallurgical reduction processes can be used for breaking the lattice [12].

The International Nickel Company (INCO) developed a pyrometallurgical process to separate valuable metals from manganese and iron in the initial stage by reduction smelting at 1000 °C in a rotary kiln in order to convert Cu, Ni, Co, and small amounts of Fe to metal and to report most of Mn and Fe to slag in an electric arc furnace at 1400 °C [13]. The metal alloys went through oxidizing, sulphidizing, and converting steps to remove Mn and Fe impurities and recover matte. The residual Fe was additionally removed from the matte, which was subsequently pressure-leached in sulfuric acid. The base metals were subjected to solvent extraction for their selective separation. Based on the INCO process, the conversion of Mn and Fe slag to ferromanganese alloy was successfully verified in the research work carried out by Sommerfeld et al. [14].

The Kennecott Copper Corporation process was based on ammoniacal leaching of the nodules in the presence of cuprous ions and CO_2_ [15]. Acting as a reducing agent, the cuprous ions reduce MnO_2_, releasing Cu, Ni, and Co into solution. Mn and Fe remain as a residue in the form of carbonate. Cupric amine complex is converted back to cuprous one by introducing CO into leaching solution. Cu, Ni, and Co were proposed to be selectively separated from leaching liquor by solvent extraction technique [16,17].

The Métallurgie Hoboken Overpelt process uses hydrochloric acid as a leachant for reductive dissolution of MnO_2_ to form MnCl_2_ and Cl_2_ [18]. The base metals released into leaching liquor were separated by solvent extraction and precipitation routes. Fe was removed by solvent extraction technique. Cu was selectively separated by sulfide precipitation using H_2_S. Ni and Co was precipitated as a mixture of NiS and CoS. Chlorine gas was utilized to oxidize manganous chloride to precipitate manganese dioxide [12]. The Deep-Sea Venture process is based on the similar route used in the Métallurgie Hoboken Overpelt process with the only difference in the treatment of generated chloride, which is converted to HCl or sold directly [19].

Selective reduction of MnO_2_ using SO_2_ was carried out in a fluidized bed reactor without presence of oxygen. The sinter was water leached to obtain manganese sulfate solution, from which manganese can be electrowon. The oxidative leaching in the presence of air and SO_2_ was used to extract Cu, Ni, and Co while iron remained in leaching residue [20].

The IMMT (Institute of Minerals and Materials Technology) process is based on high-pressure reductive ammoniacal leaching of the nodules using SO_2_ as the reducing agent [21,22,23]. Ni, Cu, and Co dissolved into leaching solution in the form of amine complexes, while Fe precipitated as ammoniacal jarosite. After demanganization by oxygen in autoclave and the recovery of ammonia, the refined solution was subjected to solvent extraction, electrowinning, and precipitation to obtain cathode copper and a mixture of NiS and CoS. 

The HZL (Hindustan Zinc Ltd., Udaipur, India) process involves pre-leaching nodules in dilute sulfuric acid at ambient temperature, followed by pressure leaching [24]. The pre-leached residue containing the valuable metals were undergone pressure leaching in sulfuric acid. After removing the main impurities (Fe, Mn, and Si) in leaching liquor, Ni, Cu, and Co were separated selectively by solvent extraction and recovered as metals by electrowinning. 

The CSIR:NML (Council of Scientific and Industrial Research—National Metallurgical Laboratory) process was developed to selective dissolution of Ni, Cu, and Co in the form of ammine complex while precipitating most of Fe and Mn in residue [25,26,27]. The process combined reduction roasting of nodule pellets in CO to produce Ni, Cu, and Co metals with two-stage ammoniacal leaching at room temperature to selectively dissolve the metals from the roasted pellets and to precipitate manganese and iron. Ni, Cu, and Co were separated from the leaching liquor by solvent extraction and recovered as metals by electrowinning.

From the facts mentioned above, it can be concluded that the processes developed and tested for the processing of deep-sea nodules are very complex, all counting with the extraction of individual metals. However, what if the natural proportions of the elements in deep-sea nodules make sense from the metallurgical point of view? When we will be able to reduce the deep-sea nodules as they are (without separation to individual metals), we could obtain completely new alloy (“natural alloy”), which could have potentially interesting properties and open new possibilities of future applications.

Aluminothermic processes [28] are based on the reaction of metal oxide with aluminum, which acts as a reducing agent. The reaction is driven by the high affinity of aluminum to oxygen. Once initiated, the aluminothermic reduction is rapid and in general strongly exothermic. The advantages of aluminothermic processes are, apart from the rapidity of the reaction, no external heat source, small plant investment costs, easy adaptation to various productions, and less gas volume. Disadvantages include no possibility of refining of the metal and relatively expensive aluminum. The process has been used for decades, especially for production of certain types of ferroalloys, where high purity is required. In the past, it also had been used for manganese production. As the major component of the nodules is manganese, aluminothermic reduction seems to be an appropriate method for obtaining an alloy from them.

In this work, we tested aluminothermic reduction of the nodules with various amounts of aluminum and characterized microstructure, phase composition, and selected properties of the new “natural alloys”.

## 2. Materials and Methods 

Deep sea nodules from the Clarion–Clipperton Zone were crushed and ground to a powder with the particle size under 125 µm. The chemical composition of the crushed powder analyzed by atomic absorption spectrometer (AAS, GBC 932plus, GBC Scientific Equipment Ltd., Dandenong, Australia) and X-ray fluorescence spectrometer (XRF, Axios, PANanalytical, Almelo, The Netherlands) is presented in Table 1. The content of Mn, Fe, Cu, Ni, Zn, and Co was measured by the AAS method.

One half of the powder amount, used subsequently for the aluminothermic reduction, was dried at 250 °C for 4 h, while the other half was annealed at 500 °C for 4 h in order to change the oxidation state of manganese in original MnO_2_ to Mn_3_O_4_. The mixture of the mentioned two states of powder was subjected to aluminothermic reduction. Aluminum was used in stoichiometric amount calculated to allow the reduction of the oxides of Mn, Fe, Cu, Ni, Zn, and Co, as could be expected on the basis of the Ellingham’s diagram [30], as well as in the excess of 10 and 20 wt. %. The aluminothermic reduction was carried out in a ceramic crucible of 85 mm in diameter; the batch was 250 g. The aluminothermic reactions were initiated by the ignition mixture composed of aluminum powder, sodium peroxide, and magnesium metal flakes. 

The metallic product of the aluminothermic reduction was separated from the slag mechanically. The reduced alloy was remelted by the means of the induction melting furnace Balzers VSG-02 (Balzers, Germany) in order to remove the slag residues and to homogenize the alloy. The obtained as-cast alloy was characterized from the viewpoints of the microstructure, phase composition, and hardness. Samples were mounted into conductive Bakelite and prepared by conventional metallography procedure for microstructure observation or grinded for the phase analysis.

Materials were investigated by scanning electron microscope (SEM) TESCAN FERA 3 (TESCAN, Brno, Czech Republic) equipped with EDAX analytical system for EDS (Energy Dispersive Spectrometer) detector Octane Super 60 mm^2^ and EBSD (electron backscatter diffraction) camera DigiView V (AMETEK, Inc., Berwyn, PA, USA). All measurements were done in combined EDS/EBSD mode to allow phase separation by composition information (CHI scan) and Kikuchi pattern to be saved for Neighbor Pattern Averaging and Reindexing (*NPAR™*).

Phase composition of materials was studied by powder X-ray diffraction (XRD). Data were collected in Bragg–Brentano geometry on a Bruker D8 Advance diffractometer using a CuKα radiation and LynxEye-XE detector. Qualitative phase analysis was performed by means of the HighScore program; subsequent semi-quantitative phase analysis and calculation of unit-cell and microstructural parameters was carried out by the Rietveld method using the Topas 5 program (Bruker AXS, Karlsruhe, Germany).

Phase transformations during heating were investigated by differential thermal analysis (DTA) by the means of TG-DTA (Thermogravimetry-Differential Thermal Analysis) Setsys Evolution device (Setaram, Caluire-et-Cuire, France) in argon atmosphere, alumina crucible, using the heating rate of 30 K/min.

Mechanical properties of the as-cast material were determined by the means of microhardness measurement. For this purpose, Vickers method with the load of 9.8 N (HV1) was applied. The hardness was measured five times on each sample. In order to determine the microhardness of individual phases, the Vickers method HV0.01 was applied. The wear resistance was measured using the ball-on-disc tribometer TriboTester (Tribotechnic, Clichy, France) in linear reciprocating mode (excenter of 5 mm), where the “ball” of 6 mm in diameter was made of alumina (α-Al_2_O_3_) and the “disc” was the sample polished to the roughness of approx. 0.005 µm. No lubrication was used. The normal force used in the test was 5 N and the sliding distance was 20 m. The wear rate was calculated from the wear track section area by Equation (1):

(1) $$w=\;\frac{A\cdot e}{F\cdot l}$$

where *w*, *A*, *e*, *F,* and *l* are wear rate [mm^3^ N^−1^ m^−1^], wear track section area [mm^2^], excenter (5 mm), normal force (5 N), and sliding distance (20 m), respectively. The wear track section area was measured by the means of a skidless contact profilometer probe (Tribotechnic, France). Each sample was measured two times, and the presented value is the average of two measurement results. Wear tracks were observed by the means of a scanning electron microscope (SEM) Vega 3 LMU (TESCAN, Brno, Czech Republic) in backscattered electrons (BSE) mode.

## 3. Results

### 3.1. Chemical Composition of the Alloys

The reduced alloys are based on manganese with major amounts of iron, silicon, and copper. There is a significant dependence of aluminum content, bound in the intermetallic phases, on the amount of aluminum used for reduction. While the alloy reduced using stoichiometric amount of aluminum contains just a minor amount of it, the others have up to more than 9 wt. % of aluminum. The iron and silicon amount is decreasing with the increasing content of aluminum used for reduction, while the other elements do not exhibit systematic trends. The chemical composition of the alloys is listed in Table 2. Among the impurities, phosphorus content is high (up to nearly 0.5 wt. %). 

### 3.2. Microstructure and Phase Composition

**Figure 1:**
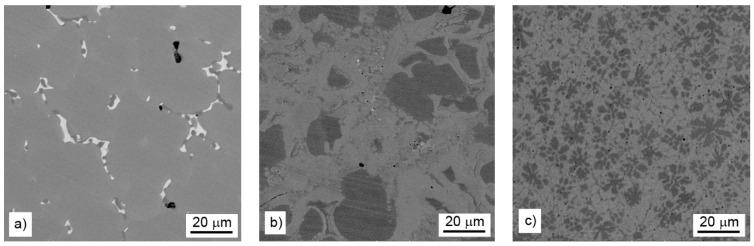
Microstructure of aluminothermically prepared samples (**a**) with stoichiometric amount of aluminum, (**b**) with 10% of aluminum excess, and (**c**) with 20% of aluminum excess.

Microstructure of the alloys is presented in Figure 1. The alloy with the lowest amount of aluminum (reduced by stoichiometric amount of aluminum, see Figure 1a) is covered mostly by a manganese-rich phase and minor amounts of secondary phases at the grain boundaries. With increasing aluminum content in the alloys, the aluminum-rich regions with round shape or flower-like morphology appear, and their fraction grows (Figure 1b,c). The phase composition and microstructure of individual alloys are described in more details below.

#### 3.2.1. Stoichiometric Amount of Aluminum

**Figure 2:**
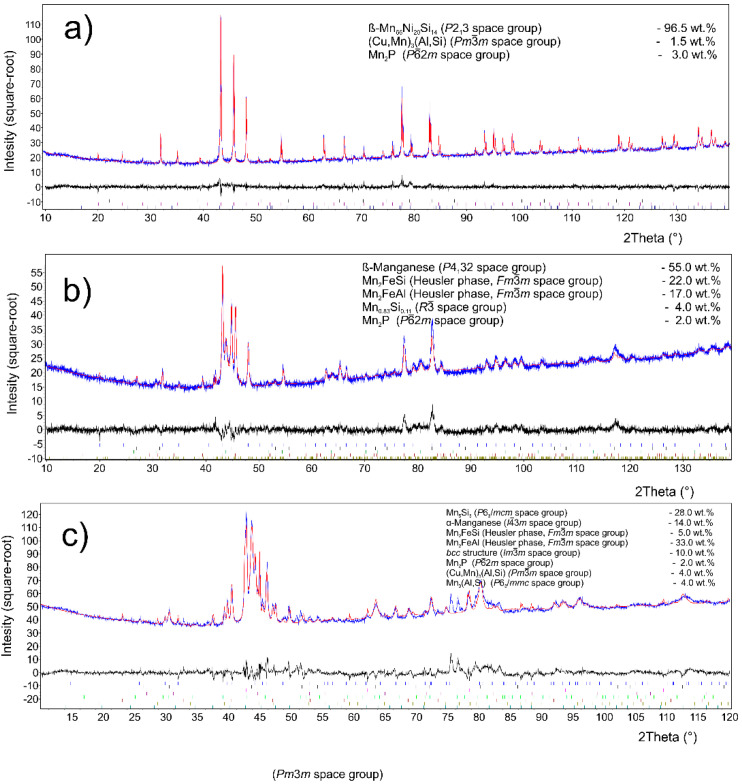
Phase composition by XRD for sample (**a**) with stoichiometric amount of aluminum; (**b**) with 10% of aluminum excess; and (**c**) with 20% of aluminum excess. The blue color indicates the measured XRD patterns, while the red color is the Rietveld fit, the black color shows the difference between the real pattern and the numerical fit.

**Figure 3:**
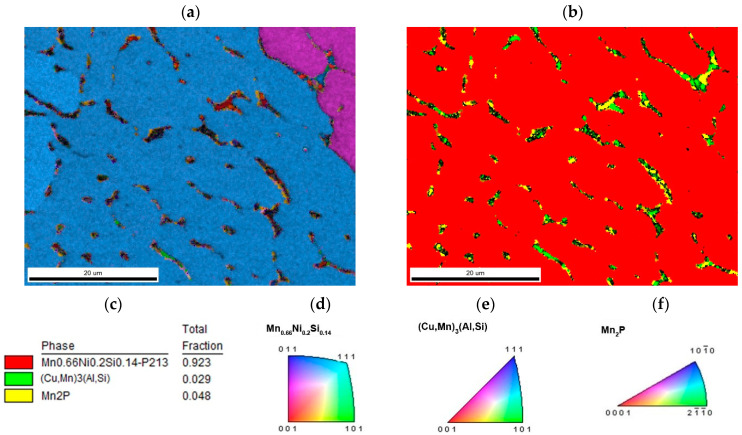
The sample with stoichiometric amount of aluminum (**a**) inverse pole figure (IPF) map overlayed on a greyscale Image Quality map; (**b**) phase distribution overlayed on a greyscale Confidence Index map, whose color code and percentage of area coverage are given in (**c**). Colorized legend of particular phases are in (**d**–**f**).

**Figure 4:**
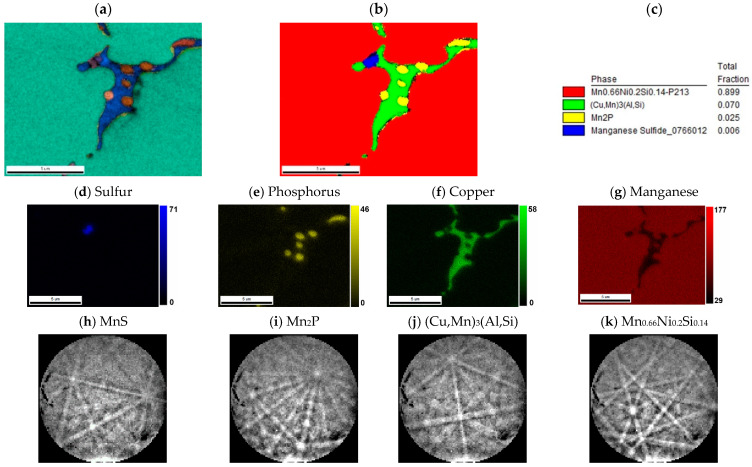
Detail of minor phases in sample with stoichiometric amount of aluminum. (**a**) IPF map overlayed on a greyscale Image Quality map; (**b**) phase distribution map overlayed on a Confidence Index map, whose color code and percentage of area coverage are given in (**c**). The middle line shows EDS results of (**d**) sulfur, (**e**) phosphorus, (**f**) copper, and (**g**) manganese distribution; scales give the intensity of respective peaks. The bottom line shows Kikuchi patterns of (**h**) MnS, (**i**) Mn_2_P, (**j**) (Cu,Mn)_3_(Al,Si), and (**k**) Mn_0.66_Ni_0.2_Si_0.14_ phases.

The major phase covers nearly 96.5 wt. % of sample weight, and its grains are separated by thin layer of minor phases, Figure 1a. The major phase corresponds to the Mn_0.66_Ni_0.2_Si_0.14_ phase (referred also as β-Mn66Ni20Si14 [31]) with *P*2_1_3 symmetry (Figure 2). According to the EDS analysis, this phase also contains a high amount of iron and minor amounts of aluminum and copper, Table 3. The crystal structure of this phase is strongly related to the structure of β-Mn, i.e., its space group (*P*2_1_3) is a subgroup of that for β-Mn (*P*4_1_32). We detected by EDS three different composition areas in minor phase—one is enriched by copper and aluminum, the other by phosphorus and even parts enriched by sulfur can be found. The first minor phase (1.5 wt. %) has space group *Pm*-3*m* and can be fitted well by both XRD and EBSD to atoms distribution as in the anti-perovskite phase Mn_3_AlC. However, the real occupation of sublattices is different—approx. 48 wt. % Cu, 36 wt. % Mn, 5 wt. % Fe, 4 wt. % Ni, and 5 wt. % Al, 1 wt. % Si. The content of non-metallic elements is too low to create sublattice—negligible amount of C and 0.7 wt. % of P, see Table 3. Thus, we can presume the minor phase is rather L1_2_ structure (Cu,Mn)_3_(Al,Si) with space group *Pm*-3*m* stabilized by copper and aluminum or by phosphorus in octahedral anti-site positions, derived from Cu_3_Al phase. The second minor phase Mn_2_P (space group *P-62m*) can be easily identified by the hexagonal structure and phosphorus content (Figure 3 and Figure 4). The third minor phase is sulfide MnS (space group *Fm-3m*) and was identified by EDS as sulfur segregate is particular particles. The amount of phase is too low to separate it from (Cu,Mn)_3_(Al,Si) diffraction pattern. 

#### 3.2.2. 10% of Aluminum Excess

**Figure 5:**
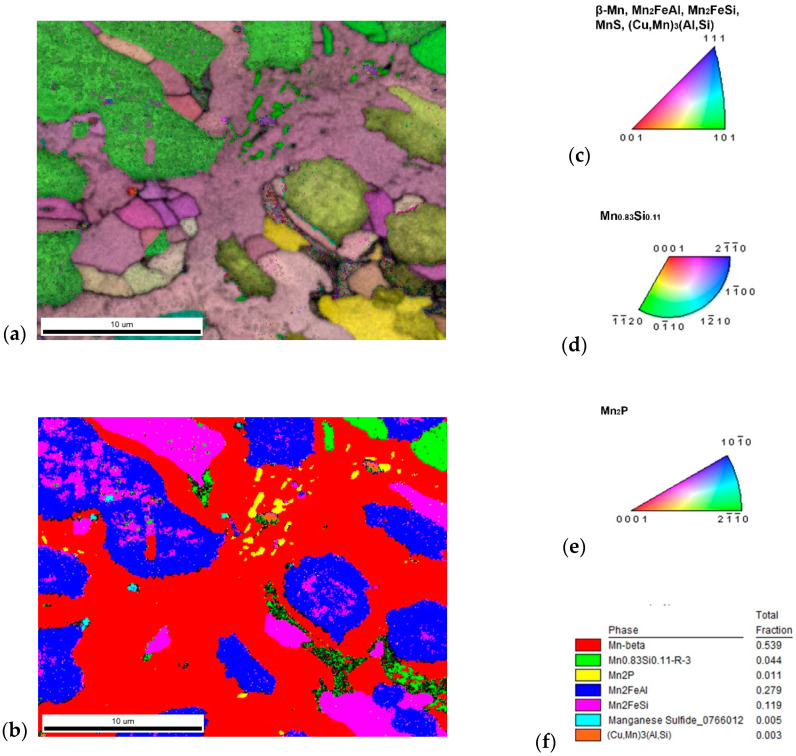
The sample with 10% excess of aluminum (**a**) inverse pole figure (IPF) map overlayed on a greyscale Image Quality map; (**b**) phase distribution overlayed on a greyscale Confidence Index map, whose color code and percentage of area coverage are given in (**f**). Colorized legend of particular phases are in (**c**–**e**).

**Figure 6:**
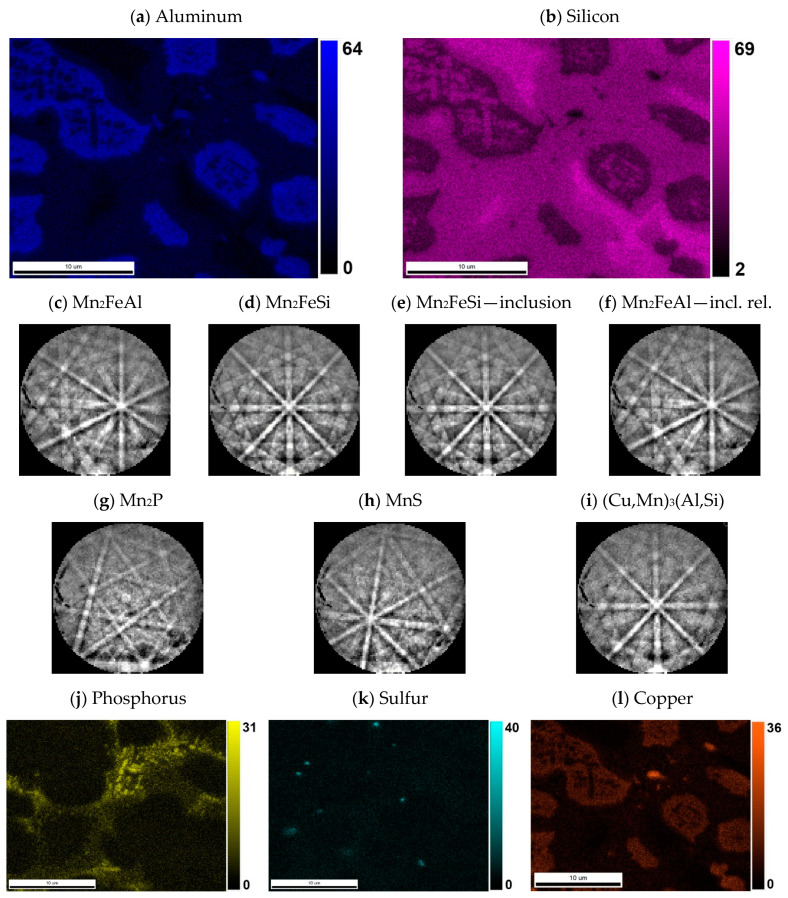
The content of (**a**) aluminum and (**b**) silicon in the sample with 10% excess of aluminum. The distribution of both elements was used for the segmentation of similar Heusler phases Mn_2_FeAl and Mn_2_FeSi. Both phases have different Kikuchi patterns: (**c**) Mn_2_FeAl has more blur bands than (**d**) Mn_2_FeSi, not only in one phase grain, but even in (**e**) inclusions of Mn_2_FeSi and close (**f**) Mn_2_FeAl matrix. Minor phases are represented by Mn_2_P (**g**) related to increased phosphorus—see (**j**), (**h**) MnS related to content of sulfur—see (**k**); and even high copper—see (**l**); content gives rise to (**i**) (Cu,Mn)_3_(Al,Si) with a different character of Kikuchi pattern.

XRD analysis indicated a β-Mn as a major phase (ca 55 wt. %) in the sample. Two phases with crystal structure of Heusler phases (*Fm*-3*m*) were also identified (Figure 2). Their chemical composition can be expressed in a simplified way as Mn_2_FeSi (22 wt. %) and Mn_2_FeAl (17 wt. %). Although these phases show the same crystal structure, they differ in the unit cell parameters (5.714 and 5.838 Å) and hence are distinguishable by XRD. Phases with a structure of Mn_0.83_Si_0.11_ (*R*-3) and Mn_2_P (*P*-62*m*) were detected as minor phases with concentration of 4 and 2 wt. %, respectively. All mentioned phases can be distinguished by EBSD (Figure 5 and Figure 6), except the structurally same cubic phases Mn_2_FeSi, Mn_2_FeAl, and MnS, which were separated by evaluation of the simultaneously acquired EDS data (CHI scan). Both Heusler phases can be separated by CHI scan only if the magnification is high enough to separate areas with higher and lower silicon or aluminum content. When such separation is possible, we can even see a different structure of Kikuchi patterns in both phases—Mn_2_FeSi have sharper Kikuchi bands. Manganese sulfide was identified by sulfur content (see Table 4 and Figure 6); Kikuchi patterns in sulfur-rich areas show cubic structure different from both Heusler phases, but without chemistry data, the separation would not be possible. Some small areas show higher copper content, such areas have distinctive Kikuchi patterns, and we assign them to Mn_3_AlC type phase, as in the case of the sample with stoichiometric amount of aluminum. Let us note that some grains of Mn_0.83_Si_0.11_ phase have bad patterns (low image quality), probably due to the polishing procedure.

#### 3.2.3. 20% of Aluminum Excess

The phase composition of this sample is more complicated in comparison with two previous samples. XRD analysis (Figure 2) revealed presence of a phase with Mn_5_Si_3_-type structure (*P*6_3_/*mcm*) and α-Mn (*I*-43*m*) in concentration 28 and 14 wt. %. Both Heusler-type alloys, Mn_2_FeAl (33 wt. %) and Mn_2_FeSi (5 wt. %), were also detected in this sample (Table 5). A phase with a *bcc* structure (*Im*3*m*) was detected in an amount of 10 wt. %, but it is limited to a minor part of the sample, as equilibrium of alloy was probably not reached yet. It corresponds to δ-Mn phase with highest Mn content. Minor compounds revealed by XRD include Mn_2_P (*P*-62*m*, 2 wt. %), phase with the L1_2_-structure (Cu,Mn)_3_(Al,Si) (4 wt. %), and Mn_3_(Al,Si) phase crystalizing in the Ni_3_Sn-structure type (*P*6_3_/*mmc*, 4 wt. %). 

**Figure 7:**
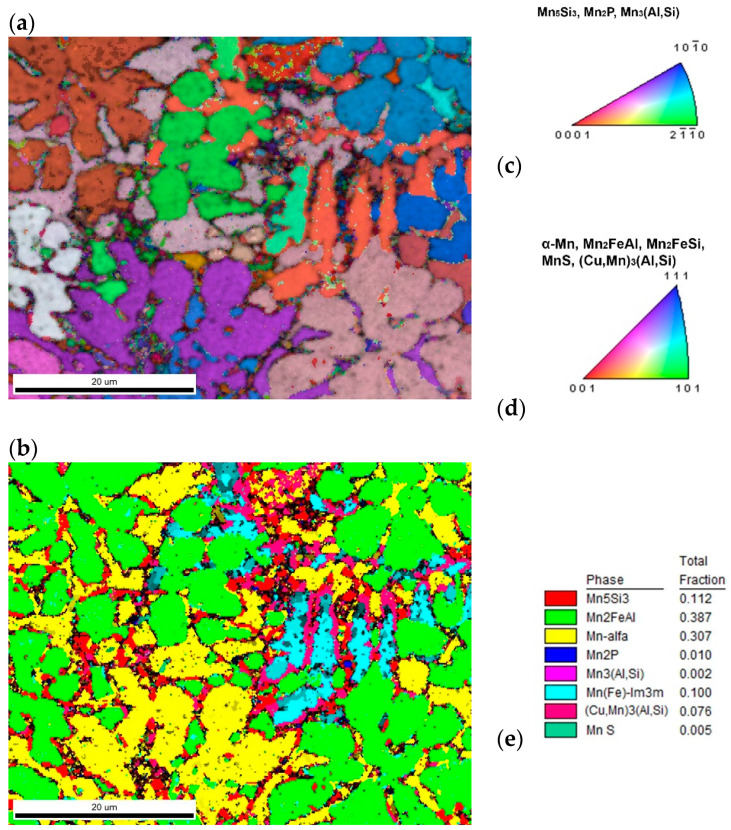
The sample with 20% excess of aluminum (**a**) inverse pole figure (IPF) map overlayed on a greyscale Image Quality map; (**b**) phase distribution overlayed on a greyscale Confidence Index map, whose color code and percentage of area coverage are given in (**e**). Colorized legends of particular phases are in (**c**,**d**).

The phases with distinct crystallographic structure were separated by Kikuchi pattern indexing as in previous cases; nevertheless, the separation by composition was more complicated here. Heusler-type alloys, Mn_2_FeAl and Mn_2_FeSi, could not be separated, as Mn_2_FeSi is minor, creating small particles in larger Mn_2_FeAl grains, and thus compositional data do not give strong enough difference for thresholding (Figure 7). On the contrary, if EBSD data were acquired with high Kikuchi pattern quality (binning 2 × 2), phase with L1_2_-structure (Cu,Mn)_3_(Al,Si) was found by pattern indexing, although not as the Cu major phase, but compositionally similar to Mn_2_P, but with *Pm-3m* space group. 

The connection of MnS with high sulfur content is less pronounced here, probably due to low quality of the simultaneously acquired EDS data. Generally, the quality of EDS data strongly affects possibility of thresholding, mainly for small particles, where overlap of compositional signal with surrounding grains is expected. 

### 3.3. Differential Thermal Analysis

**Figure 8:**
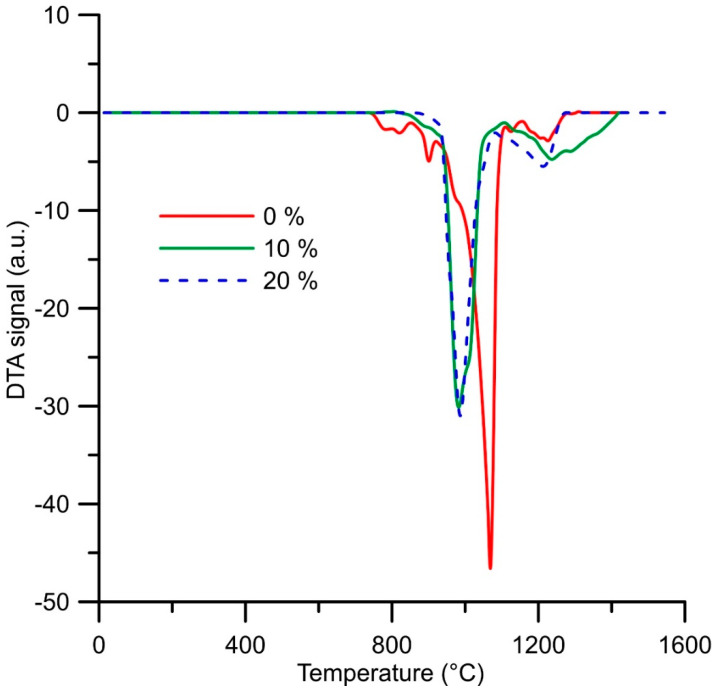
DTA heating curves of the alloys (in wt. %) prepared by reduction with a stoichiometric amount of aluminum (0%), with 10% excess of aluminum (10%) and with 20% excess of aluminum (20%).

Thermal properties have been studied in order to determine the phase transformations during heating. It was found that the alloy reduced using the stoichiometric amount of aluminum undergoes a series of minor endothermic phase transformations between 750 and 930 °C (Figure 8). These transformations are probably connected with the transformation of some minor phases in the structure. After that, a strong endothermic effect is observed with the onset temperature of approx. 930 °C. This effect probably accompanies the eutectic transformation. The next endothermic effect is visible at approx. 1150 °C, belonging probably to the liquidus temperature of the alloy.

In the case of both alloys reduced with the excess amount of aluminum, there are two endothermic effects at very similar temperatures—the first one (probably the eutectic transformation) at approx. 910 °C and the second one (probably the liquidus) at 1140 °C for the alloy reduced with 10% excess of aluminum and 1100 °C for the alloy reduced using the 20% excess of aluminum (Figure 8). Since the significant thermal effect occurs at the same temperature, it can be expected that it accompanies the eutectic reaction between the Heusler phases, which were identified in both alloys with the excess amount of aluminum.

### 3.4. Mechanical and Tribological Properties

Hardness of the tested alloys reaches 732 ± 15, 790 ± 19, and 813 ± 10 HV1 for alloy reduced using stoichiometric amount, 10% and 20% excess of aluminum, respectively. The aluminum-rich areas are softer (718 HV0.01) than the manganese- and silicon-rich one (828 HV0.01), as determined in the alloy prepared with the 20% excess of aluminum. 

**Figure 9:**
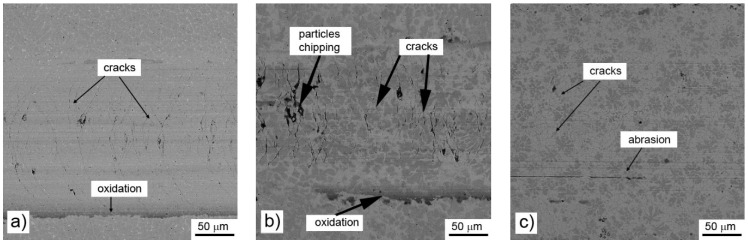
Wear tracks on aluminothermically prepared samples after testing with Al_2_O_3_ ball as the static partner: (**a**) alloy reduced with stoichiometric amount of aluminum; (**b**) with 10% of aluminum excess and (**c**) with 20% of aluminum excess.

All tested alloys also reached almost the same value of the friction coefficient (0.67–0.68) during the wear test against the alumina ball (Table 6). The reached value is comparable with common heat-treated steel and other comparable materials [32]. The wear rate of the alloys is increasing with the growing amount of aluminum in the material (Table 6) and reaches the values comparable with heat-treated AISI D2 and Fe-Al-Si intermetallics (in the order of 10^−6^ mm³ N^−1^m^−1^) [33]. When observing the worn surfaces by scanning electron microscope, it can be seen that the wear mechanism changes gradually. In the case of the material prepared with stoichiometric amount of aluminum, there are almost no signs of a chipping wear. The observed wear is abrasive, with negligible signs of oxidation (Figure 9a). On the other hand, the cracks perpendicular to the sliding direction could be observed. These cracks are probably caused by the accumulation of the heat in the sample. Due to relatively high friction coefficient, the contact area is getting heated. The thermal conductivity of the alloy is expected to be lower than that of steel, because of the low thermal conductivity of manganese, and so the heat accumulates in worn area. The local thermal gradient causes cracking of the material in the area of the wear track. A similar situation can be observed in the alloy prepared with the 10% excess of aluminum, where the dominant phase is still derived from β-Mn (Figure 9b). In this sample, the cracks are less significant. In addition to the cracks, the chipping of particles of aluminum-rich phase could be observed locally. In the case of the sample with the highest amount of aluminum, the wear mechanism changes. There are only minor cracks present locally, being initiated in the Mn-rich phase, which do not propagate to softer Al-rich phase. The wear track is more abraded than in the previously described samples (Figure 9c). It is probably caused by lower hardness of the Al-rich phase.

**Figure 10:**
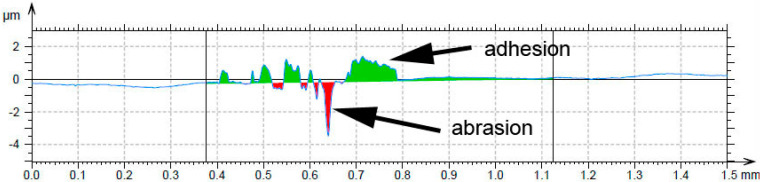
Wear track profile on the alloy reduced with stoichiometric amount of aluminum after testing by steel 100Cr6 ball.

**Figure 11:**
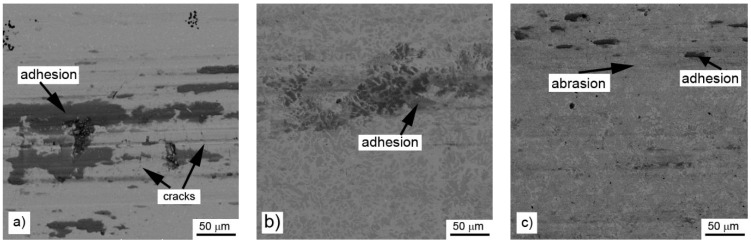
Wear tracks on aluminothermically prepared samples after testing with 100Cr6 steel ball as the static partner: (**a**) alloy reduced with stoichiometric amount of aluminum; (**b**) with 10% of aluminum excess and (**c**) with 20% of aluminum excess.

When the wear behavior was tested by the use of a steel ball (100Cr6) as the static partner, the achieved friction coefficients were varying with the aluminium content, showing no systematic trend, see Table 6. It is probably caused by random adhesion of the steel ball residues on the surface of the tested materials, as it was proved by the measurement of the profile of the wear track (Figure 10) and by the observation of the worn surfaces by SEM (Figure 11). There are far fewer cracks formed on the sample reduced with stoichiometric amount of aluminum (Figure 11) than after testing with the alumina ball and no cracks in the samples with higher amounts of aluminum. This confirms that the formation of the wear tracks is caused by the thermal shocks due to the evolution of heat by the dissipation of the friction energy. In the case of the use of a more thermally conductive static friction partner (100Cr6 steel), the heat dissipates more easily from the wear track.

## 4. Discussion

The alloys prepared by aluminothermic reduction with various amounts of aluminum are characterized by a very rich phase composition. In addition, the particular phases are not listed in current version of the PDF database, and hence the results can deepen the knowledge about crystallography of manganese-rich intermetallics. A very interesting point in the phase composition is the fact that the matrix phase changes, as the content of aluminum in the alloy increases. It varies from Mn_0.66_Ni_0.2_Si_0.16_ (structure similar to β-Mn) in the case of the alloy prepared with stoichiometric amount of aluminum through the β-Mn solid solution in the alloy prepared with 10% excess of aluminum and ends with α-Mn in the highest-aluminum alloy. At first sight, this variation is strange, because aluminum stabilizes the β-Mn phase and the aluminum content in the matrix increases with growing amount of aluminum used for reduction. The probable reason is that the amounts of nickel and copper in the Mn-based matrix tend to decrease, as the amount of aluminum used for reduction increases from 10 to 20% excess (see Table 4 and Table 5), because they are bound in the aluminum-rich phases. Nickel is the stabilizer of the beta phase, as it can be seen in the Mn–Ni binary equilibrium phase diagram [34]. 

The next paradox found during the characterization of the materials is connected with the hardness. The hardness keeps almost constant, even though the aluminum-rich phases, whose fraction increases with the amount of aluminum used for reduction, are softer than the manganese-rich matrix. The reason is the increasing content of aluminum dissolved in the matrix. Aluminum could cause the local stresses in the lattice due to a different atomic radius [35], leading to the strengthening effect.

Even though there is almost no effect of aluminum content on the friction coefficient, the wear resistance and the wear mechanism are strongly dependent on it, especially in the case of the use of a static partner with low thermal conductivity (alumina ball). In this case, the wear mechanism changes from the abrasive wear with the significant presence of cracks in the case of the alloy prepared by reduction using the stoichiometric amount of aluminum through chipping wear with strong cracks to purely abrasive wear with a low number of short cracks. These cracks stop in the aluminum-rich phase, which is softer and probably more ductile than the manganese-based matrix. For the same reason, the abrasive wear is most intense in the case of the aluminum-rich phase in the case of high-aluminum alloy. When the static partner is well thermally conductive (steel ball), the formation of cracks is minimized. It shows that the cracks originate from the heat caused by the dissipation of the friction energy. From this fact and the low wear rates, it can be derived that the materials, especially the high-aluminum ones, are suitable for use as wear resistant materials for the metal–metal contact applications. 

The practical consequences of thermal analysis for the processing are the following: If the material would be processed by melting and casting, then the melting temperature should be higher than 1200 °C. If powder metallurgy will be considered as the processing route, solid-phase sintering has to be done below 900 °C for the alloys with higher contents of aluminum and less than 750 °C for the alloy reduced using the stoichiometric amount of aluminum.

## 5. Conclusions

It has been proven that the aluminothermic reduction can be used for processing of deep-sea nodules. The process leads to obtaining completely new manganese-based alloys. The phase composition of the alloy prepared using the stoichiometric amount of aluminum consists mainly of Mn_0.66_Si_0.20_Si_0.14_ type phase (structure similar to β-Mn), (Cu,Mn)_3_(Al,Si) phase, and Mn_2_P. In the case of the reduction with the aluminum excess of 10%, the sample consisted mostly of β-Mn type phase and Heusler phases of Mn_2_FeSi and Mn_2_FeAl types. The alloy reduced using the 20% excess of aluminum is based on both mentioned Heusler phases, α-Mn type phase and Mn_5_Si_3_ silicide.

The alloys are characterized by relatively high hardness (around 800 HV) and very good wear resistance, especially when high amount of aluminum (20% over stoichiometry) is used for reduction. The lower-aluminum alloys tend to form cracks during wear testing against the alumina ball. The tested materials can be processed by induction melting and gravity casting and can be recommended as wear resistant materials for the metal–metal sliding contacts, especially the high-aluminum one, which does not tend to exhibit local cracking in the contact area.

## Figures and Tables

**Table 1:** Chemical composition (XRF) of the ground deep-sea nodules (wt. %) [29].

Element	Mn	Fe	Si	Al	Mg	Ca	Na	Cu	Ni	Ti	Zn	Co	O
wt. %	30.57	4.41	3.53	2.16	1.87	1.84	1.64	1.18	1.14	0.35	0.14	0.13	bal.

**Table 2:** Chemical composition of the alloys (in wt. %) prepared by reduction with a stoichiometric amount of aluminum, with 10% excess of aluminum and with 20% excess of aluminum.

	Stoichiometric Al	10% Excess Al	20% Excess Al
Mn	57.478	52.613	57.708
Al	0.845	5.582	9.312
Si	8.54	8.289	8.001
P	0.457	0.257	0.487
S	0.037	0	0.118
V	0.173	0.175	0.236
Fe	20.717	19.606	15.361
Co	0.536	0.586	0.545
Ni	6.427	6.675	4.083
Cu	4.535	5.949	3.935
Mo	0.255	0.268	0.214

**Table 3:** Composition in at. % of phases in sample with stoichiometric amount of aluminum measured by EDS. Measured compositions from areas investigated by EBSD are given. Error of measurement depends on number of counts in peak. The elements typical for minor phases are given in bold.

Element	Mn_0.66_Ni_0.2_Si_0.14_	(Cu,Mn)_3_(Al,Si)	Mn_2_P	MnS
Al K	1.7	9.48	1.86	2.17
Si K	13.98	2.53	7.62	4.13
P K	0.05	1.16	10.11	0.37
S K	0.08	0.14	0.05	20.68
Ti K	0.07	0.24	0.29	0.38
Mn K	59.98	36.04	57.74	53.96
Fe K	18.36	4.99	8.08	6.37
Ni K	3.75	3.98	2.71	2.13
Cu K	2.03	41.44	11.54	9.81

**Table 4:** Composition in at. % of phases in sample with 10% excess of aluminum measured by EDS. Measured compositions from areas investigated by EBSD are given. Error of measurement depends on number of counts in peak.

Element	β-Mn	Mn_0.83_ Si_0.11_	Mn_2_P	Mn_2_FeAl	Mn_2_FeSi	MnS	(Cu,Mn)_3_(Al,Si)
Al K	6.09	4.83	2.64	18.28	2.45	7.5	15.72
Si K	14.02	15.91	11.97	8.72	18.4	10.94	5.28
P K	0.94	0.46	7.53	0	0.06	1.42	1.94
S K	0	0	0.01	0	0	1	0
Ti K	0.42	0.5	0.87	0.11	0.13	2.28	0.42
Mn K	57.13	58.39	59.28	46.24	61.74	56.6	38.07
Fe K	15.62	16.72	14.39	14.51	16.67	14.65	9.41
Ni K	3.26	3.19	2.11	5.28	0.29	2.94	5.44
Cu K	2.52	1.41	1.2	6.86	0.26	2.67	23.72

**Table 5:** Composition of phases in sample with 20% excess aluminum measured by EDS. Measured compositions from areas investigated by EBSD are given. Error of measurement depends on number of counts in peak.

Element	Mn_5_Si_3_	Mn_2_FeAl	α-Mn	Mn_2_P	Mn_3_(Al,Si)	Mn(Fe)	(Cu,Mn)_3_(Al,Si)	MnS	Ti-Part
Al K	9.36	25.06	6.76	8.27	9.45	16.03	9.82	6.69	6.65
Si K	15.32	5.43	13.95	14.57	15.06	3.56	14.41	12.03	11.38
P K	0.20	0.04	0.06	3.59	1.65	0.20	3.57	1.21	0.54
S K	0.14	0.07	0.11	0.15	0.29	0.14	0.09	6.43	5.58
Ti K	0.79	0.57	0.76	0.72	0.85	0.54	0.36	5.62	13.48
Mn K	55.50	40.93	61.00	54.69	52.83	68.36	55.7	54.5	47.91
Fe K	15.74	16.61	14.16	14.25	13.21	9.65	11.63	10.68	11.01
Ni K	2.95	5.76	1.83	1.84	3.40	0.70	1.48	1.24	1.76
Cu K	1.98	5.53	1.37	1.92	3.26	0.82	2.94	1.60	1.69

**Table 6:** Tribological properties of tested alloys against Al_2_O_3_ and steel (100Cr6) balls: f—friction coefficient, w—wear rate.

Alloy	f (Al_2_O_3_) (-)	w (Al_2_O_3_) (mm³N^−1^ m^−1^)	f (Steel) (-)
stoichiometric Al	0.68	1.4 × 10^−6^	0.64
10% excess Al	0.68	1.9 × 10^−6^	0.76
20% excess Al	0.67	5.1 × 10^−6^	0.67

## Data Availability

The data are stored by the authors of the paper, not available publically.

## References

[1] Haynes B.W., Law S.L., Barron D.C., Kramer G.W., Maeda R., Magyar M.J. (1985). Pacific manganese nodules: Characterization and processing. Bulletin/US Dept. of the Interior, Bureau of Mines.

[2] Hein J., Mizell K., Koschinsky A., Conrad T.A. (2013). Deep-ocean mineral deposits as a source of critical metals for high- and green-technology applications: Comparison with land-based deposits. Ore Geol. Rev..

[3] Mohwinkel D., Kleint C., Koschinsky A. (2014). Phase associations and potential selective extraction methods for selected high-tech metals from ferromanganese nodules and crusts with siderophores. Appl. Geochem..

[4] Randhawa N.S., Hait J., Jana R.K. (2016). A brief overview on manganese nodules processing signifying the detail in the Indian context highlighting the international scenario. Hydrometallurgy.

[5] Sen P.K. (1999). Processing of sea nodules: Current status and future needs. Met. Mater. Process..

[6] Sen P.K. (2010). Metals and materials from deep sea nodules: An outlook for the future. Int. Mater. Rev..

[7] Cardwell P.H. (1973). Extractive metallurgy of ocean nodules. Min. Cong. J..

[8] Hubred G.L. (1980). Manganese nodule extractive metallurgy. A review. Mar. Min..

[9] Premchand, Jana R.K. Processing of polymetallic sea nodules: An overview. Proceedings of the Third ISOPE-Ocean Mining Symposium (OMS).

[10] Vu H., Jandová J., Lisá K., Vranka F. (2005). Leaching of manganese deep ocean nodules in FeSO_4_–H_2_SO_4_–H_2_O solutions. Hydrometallurgy.

[11] Vu H., Jandová J., Lisá K., Vranka F. (2005). Separation of copper and cobalt–nickel sulphide concentrates during processing of manganese deep ocean nodules. Hydrometallurgy.

[12] Monhemius A.J., Burkin A.R. (1980). The extractive metallurgy of deep-sea manganese nodules. Topics in Non-Ferrous Extractive Metallurgy—Critical Reports on Applied Chemistry Volume 1.

[13] Sridhar R., Jones W.E., Warner J.S. (1976). Extraction of copper, nickel and cobalt from sea nodules. J. Met..

[14] Sommerfeld M., Friedmann D., Kuhn T., Friedrich B. (2018). “Zero-Waste”: A Sustainable Approach on Pyrometallurgical Processing of Manganese Nodule Slags. Minerals.

[15] Szabo L.J. (1976). Recovery of Metal Values from Manganese Deep Sea Nodules Using Ammoniacal Cuprous Leach Solutions. U.S. Patent.

[16] Agarwal J.C. (1976). A new fix on metal recovery from sea nodules. Eng. Min. J..

[17] Skarbo R.R., Galin W.E., Natwig D.L. (1975). Cobalt Stripping from Oximes. U.S. Patent.

[18] Van Peteghem A.L. (1977). Extracting Metal Values from Manganiferrous Ocean Nodules. U.S. Patent.

[19] Kane W.S., Cardwell P.H. (1974). Process for Recovering Manganese from Its Ores. U.S. Patent.

[20] Cardwell P.H., Kane W.S. (1976). Method for Separating Metal Constituents from Ocean Floor Nodules. U.S. Patent.

[21] Acharya S., Das R.P. (1987). Kinetics and mechanism of reductive ammonia leaching of ocean nodules by manganese ion. Hydrometallurgy.

[22] Acharya R., Ghosh M.K., Anand S., Das R.P. (1999). Leaching of metals from Indian Ocean nodules in SO_2_–H_2_O–H_2_SO_4_–(NH_4_)_2_SO_4_ medium. Hydrometallurgy.

[23] Nathsarma K.C., Rout P.C., Sarangi K. (2013). Manganese precipitation kinetics and cobalt adsorption on MnO_2_ from the ammoniacal ammonium sulfate leach liquor of Indian Ocean manganese nodule. Hydrometallurgy.

[24] Basu A.K. (1989). Metallurgy of polymetallic sea nodules for recovery of value metals. Proceedings of the National Seminar on Chemical and Allied Materials from the Ocean.

[25] Srikanth S., Alex T.C., Agrawal A., Premchand Reduction roasting of deep-sea manganese nodules using liquid and gaseous reductants. Proceedings of the Second ISOPE-Ocean Mining Symposium (OMS).

[26] Jana R.K., Pandey B.D., Premchand (1999). Ammoniacal leaching of roast reduced deep-sea manganese nodules. Hydrometallurgy.

[27] Puvvada G.V.K., Jana R.K., Pandey B.D., Bagchi D., Kumar V., Premchand P. Ammoniacal leach and solvent extraction for the recovery of valuable metals from roast-reduced polymetallic Ocean nodules. Proceedings of the Second ISOPE-Ocean Mining Symposium (OMS).

[28] Hall F.W. (2000). Aluminothermic Processes. Ullmann’s Encyclopedia of Industrial Chemistry.

[29] Novák P., Vlášek J., Dvořák P., Školáková A., Nová K., Knaislová A. (2020). Microstructure of the Alloys Prepared by Reduction of Deep Sea Nodules by Aluminium and Silicon. Manuf. Technol..

[30] Seetharaman S. (2014). Treatise on Process Metallurgy, Volume 3: Industrial Processes.

[31] Shoemaker C.B., Shoemaker D.P., Hopkins T.E., Yindepit S. (1978). Refinement of the structure of [beta]-manganese and of a related phase in the Mn-Ni-Si system. Acta Cryst..

[32] Coefficient of Friction Equation and Table Chart. https://www.engineersedge.com/coeffients_of_friction.htm.

[33] Novák P., Vanka T., Nová K., Stoulil J., Průša F., Kopeček J., Haušild P., Laufek F. (2019). Structure and Properties of Fe–Al–Si Alloy Prepared by Mechanical Alloying. Materials.

[34] Massalski T.B. (1990). Binary Alloy Phase Diagrams.

[35] Atomic Radius of the Elements. https://periodictable.com/Properties/A/AtomicRadius.v.html.

